# Efficacy of umeclidinium/vilanterol according to the degree of reversibility of airflow limitation at screening: a post hoc analysis of the EMAX trial

**DOI:** 10.1186/s12931-021-01859-w

**Published:** 2021-10-28

**Authors:** Claus F. Vogelmeier, Paul W. Jones, Edward M. Kerwin, Isabelle H. Boucot, François Maltais, Lee Tombs, Chris Compton, David A. Lipson, Leif H. Bjermer

**Affiliations:** 1 Department of Medicine, Pulmonary and Critical Care Medicine, University Medical Centre Giessen and Marburg, Philipps-Universität Marburg, German Centre for Lung Research (DZL), Baldingerstraße, 35043 Marburg, Germany; 2 GSK, Brentford, Middlesex UK; 3 Altitude Clinical Consulting and Clinical Research Institute of Southern Oregon, Medford, OR USA; 4 Centre de Pneumologie, Institut Universitaire de Cardiologie et de Pneumologie de Québec, Université Laval, Québec, Québec Canada; 5 Precise Approach Ltd, GSK, Brentford, Middlesex UK; 6 Respiratory Clinical Sciences, GSK, Collegeville, PA USA; 7 Perelman School of Medicine, University of Pennsylvania, Philadelphia, PA USA; 8 Respiratory Medicine and Allergology, Lund University, Lund, Sweden

**Keywords:** Bronchodilator reversibility, COPD, Dual bronchodilators, E-RS, Lung function, Rescue medication, SAC-TDI, Umeclidinium/vilanterol

## Abstract

**Background:**

In patients with chronic obstructive pulmonary disease (COPD), the relationship between short-term bronchodilator reversibility and longer-term response to bronchodilators is unclear. Here, we investigated whether the efficacy of long-acting bronchodilators is associated with reversibility of airflow limitation in patients with COPD with a low exacerbation risk not receiving inhaled corticosteroids.

**Methods:**

The double-blind, double-dummy EMAX trial randomised patients to umeclidinium/vilanterol 62.5/25 µg once daily, umeclidinium 62.5 µg once daily, or salmeterol 50 µg twice daily. Bronchodilator reversibility to salbutamol was measured once at screening and defined as an increase in forced expiratory volume in 1 s (FEV_1_) of ≥ 12% and ≥ 200 mL 10−30 min post salbutamol. Post hoc, fractional polynomial (FP) modelling was conducted using the degree of reversibility (mL) at screening as a continuous variable to investigate its relationship to mean change from baseline in trough FEV_1_ and self-administered computerised-Transition Dyspnoea Index (SAC-TDI) at Week 24, Evaluating Respiratory Symptoms-COPD (E-RS) at Weeks 21–24, and rescue medication use (puffs/day) over Weeks 1–24. Analyses were conducted across the full range of reversibility (−850–896 mL); however, results are presented for the range −100–400 mL because there were few participants with values outside this range.

**Results:**

The mean (standard deviation) reversibility was 130 mL (156) and the median was 113 mL; 625/2425 (26%) patients were reversible. There was a trend towards greater improvements in trough FEV_1_, SAC-TDI, E-RS and rescue medication use with umeclidinium/vilanterol with higher reversibility. Improvements in trough FEV_1_ and reductions in rescue medication use were greater with umeclidinium/vilanterol compared with either monotherapy across the range of reversibility. Greater improvements in SAC-TDI and E-RS total scores were observed with umeclidinium/vilanterol versus monotherapy in the middle of the reversibility range.

**Conclusions:**

FP analyses suggest that patients with higher levels of reversibility have greater improvements in lung function and symptoms in response to bronchodilators. Improvements in lung function and rescue medication use were greater with umeclidinium/vilanterol versus monotherapy across the full range of reversibility, suggesting that the dual bronchodilator umeclidinium/vilanterol may be an appropriate treatment for patients with symptomatic COPD, regardless of their level of reversibility.

**Supplementary Information:**

The online version contains supplementary material available at 10.1186/s12931-021-01859-w.

## Background

In chronic obstructive pulmonary disease (COPD) a widely accepted definition of reversibility is a ≥ 12% and ≥ 200 mL improvement from pre-bronchodilator baseline in forced expiratory volume in 1 s (FEV_1_) within 60 min of inhalation of a single or a combination of short-acting bronchodilators (short-acting muscarinic antagonists [SAMA], short-acting β_2_-agonists [SABA]), or SAMA + SABA) [1, 2]. However, this definition is somewhat arbitrary and there is some disagreement regarding the use of this cut-off point [3]. There is also no clear relationship between the degree of bronchodilator reversibility and longer-term response to bronchodilators and it is generally thought that reversibility status should not be considered when making treatment decisions for maintenance medications in COPD [4]. Moreover, the degree of reversibility of airflow limitation is no longer recommended to differentiate COPD diagnosis from asthma [4, 5].

Although the magnitude of response and responder rates for lung function and health status improvements have been shown to be greatest in patients that met reversibility criteria, previous studies have shown patients with COPD respond to long-acting bronchodilators irrespective of their degree of reversibility [6–9]. For example, the 4-year UPLIFT trial showed no evidence of an association between baseline short-acting bronchodilator reversibility and long-term response to tiotropium in terms of lung function, St George’s Respiratory Questionnaire (SGRQ) and exacerbation risk [9]. This was also the case for the GEM and FLIGHT studies, which showed that patient-reported outcomes (SGRQ, COPD Assessment Test [CAT] and Transition Dyspnoea Index [TDI] scores, and daily rescue medication use) improvement from baseline were not associated with patient reversibility status [6, 7].

The Early MAXimisation of bronchodilation for improving COPD stability (EMAX) trial enrolled patients with symptomatic COPD with a low exacerbation risk who were not receiving inhaled corticosteroids (ICS) [10]. The primary analysis of the EMAX trial demonstrated that umeclidinium/vilanterol (UMEC/VI) dual treatment consistently provided improvements in lung function and symptoms compared with UMEC or salmeterol (SAL) monotherapy [10]. The aim of this post hoc analysis of the EMAX trial was to investigate whether the degree of bronchodilation reversibility before the treatment phase of the study (at screening) is associated with the efficacy of UMEC/VI and monotherapy in this patient population. Unlike other studies that have used arbitrary cut-off points to define which patients are considered reversible, this analysis assessed treatment response across a wide range of bronchodilator reversibility by employing fractional polynomial (FP) modelling of the degree of reversibility to salbutamol, rather than by dichotomising the data using pre-defined cut-off points.

A plain language summary of this analysis is provided in Additional file 1.

## Methods

### Study design and patients

The EMAX trial (NCT03034915; GSK study number 201749) was a multicentre, randomised, double-blind, double-dummy trial. Patients were randomised 1:1:1 to once-daily UMEC/VI 62.5/25 µg via the ELLIPTA inhaler, once-daily UMEC 62.5 µg versus ELLIPTA, or twice-daily SAL 50 µg via the DISKUS inhaler, for 24 weeks [10]. Full details of the study design and patient population have been described previously [10]. Briefly, patients were ≥ 40 years of age with a COPD diagnosis (American Thoracic Society [ATS]/European Respiratory Society [ERS] definition), were current/former smokers (≥ 10 pack-years smoking history), had a pre- and post-salbutamol FEV_1_/forced vital capacity ratio of < 0.7, post-salbutamol FEV_1_ of ≥ 30 to ≤ 80% predicted, a CAT score of ≥ 10, with ≤ 1 moderate exacerbation and had no severe exacerbations in the past year. Patients with a current diagnosis of asthma were excluded. Before screening and during the 4-week run-in period, bronchodilator maintenance therapy was limited to none or one long-acting bronchodilator (a long-acting muscarinic antagonist [LAMA] or a long-acting β_2_-agonist [LABA]). Patients were required to be free of ICS and ICS/LABA for ≥ 6 weeks and free of LAMA/LABA for ≥ 2 weeks prior to run-in. As-needed salbutamol was permitted throughout the study.

Reversibility was measured once at screening; post-bronchodilator spirometry was performed 10 to 30 min after 4 inhalations of the SABA salbutamol (total 400 µg). Patients with ≥ 12% and ≥ 200 mL improvements from pre-bronchodilator baseline in FEV_1_ were categorised as reversible for comparisons of baseline characteristics.

The study was performed according to the Declaration of Helsinki and received appropriate ethical approval. All patients provided written informed consent at their pre-screening or screening visit.

### Outcomes

Trough FEV_1_ at Week 24 was defined as the mean of the FEV_1_ values obtained 23 and 24 h after dosing on the previous day (Day 167). Patient-reported symptom assessments included self-administered computerised-TDI (SAC-TDI) at Week 24, evaluating respiratory symptoms-COPD (E-RS) total score at Weeks 21–24, and daily rescue salbutamol use (puffs/day) at Weeks 1–24.

### Statistical analyses

Results are presented for the intent-to-treat (ITT) population, consisting of all randomised patients who received ≥ 1 dose of study treatment. All analyses were conducted post hoc.

**Fig. 1:**
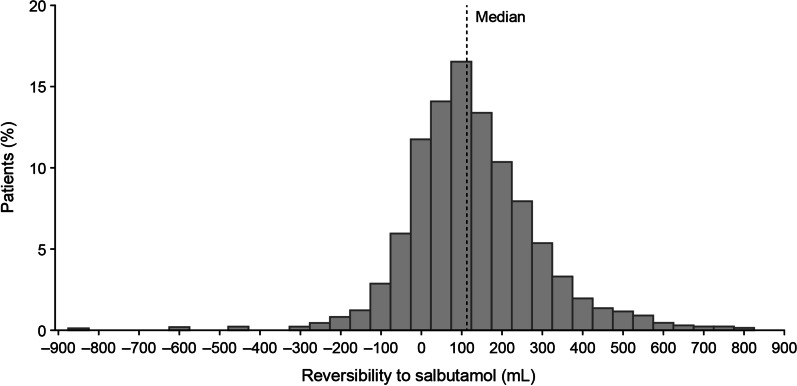
Distribution of reversibility (mL) at screening

FP modelling used the degree of reversibility to salbutamol as a continuous variable (in mL). Analyses were performed across the full range of baseline reversibility (−850 mL to 896 mL); however, due to the distribution of reversibility in the ITT population (91% [2202/2425] of patients had reversibility between −100 mL and 400 mL; Fig. 1), only data for the range −100 mL to 400 mL is shown. The best fitting FP model from the FP(2) class is presented. The fitted mixed model repeated measures (MMRM) included covariates of baseline FEV_1_, geographical region, number of bronchodilators per day during run-in, visit, treatment, FP1, FP2, and visit by baseline, visit by treatment, FP1*treatment, and FP2*treatment interactions. FP1 and FP2 represent continuous power transformations of reversibility to salbutamol at screening. Data are presented as least squares mean treatment differences from baseline, with estimated treatment differences and 95% confidence intervals (CI). No statistical tests were conducted due to the exploratory nature of the analyses.

Reversibility to salbutamol was not included as a covariate in the primary analysis of the EMAX trial; as such, its impact was not investigated [10]. Therefore, the effect of reversibility and the interaction of reversibility*treatment on trough FEV_1_, SAC-TDI focal score, E-RS total score and daily rescue salbutamol use (puff/day) were explored using MMRM with covariates of baseline FEV_1_, treatment, geographical region, number of bronchodilators per day during run-in, visit, baseline FEV_1_ reversibility, visit by baseline, treatment by baseline, visit by treatment, visit by baseline FEV_1_ reversibility and treatment by baseline FEV_1_ reversibility interactions, where visit is nominal.

## Results

### Baseline characteristics

At screening, the mean [standard deviation (SD)] reversibility to salbutamol was 130 mL (156) and the median (interquartile range [IQR]) was 113 mL (31–214). The mean (SD) percent reversibility was 10.5% (13.1) and the median (IQR) was 8.2% (2.2–16.1). Of the 2425 patients in the ITT population, 625 (26%; UMEC/VI: 212 [26%], UMEC: 207 [26%], SAL: 206 [25%]) patients met the reversibility criteria.

In the ITT population patient demographics and baseline characteristics were similar between treatment groups [10]. Of the reversible patients, 32% were female compared with 44% in the non-reversible group, and 40% versus 28% were maintenance treatment naïve, respectively (Table 1). Mean (SD) rescue medication use was 2.7 (2.9) puffs/day and 2.0 (2.3) puffs/day in reversible and non-reversible patients, respectively, and E-RS total scores were 11.7 (5.9) and 10.2 (5.6), respectively.

**Table 1:** Patient demographics and baseline characteristics in reversible and non-reversible patients at screening

Characteristic	ITT (N = 2425)	Reversible (N = 625)	Non-reversible (N = 1799)
Age, years, mean (SD)	64.6 (8.5)	63.5 (8.4)	65.0 (8.5)
Female, n (%)	988 (41)	200 (32)	788 (44)
No maintenance medication during run-in, n (%)	749 (31)	250 (40)	499 (28)
Moderate COPD exacerbation in prior year^a^, n (%)	393 (16)	84 (13)	309 (17)
Duration of COPD, years, mean (SD)	8.3 (6.6)	8.4 (6.0)	8.3 (6.8)
Post-salbutamol FEV_1_, mL, mean (SD)	1595 (511)	1455 (478)	1502 (529)
Post-salbutamol % predicted FEV_1_, mean (SD)	55.4 (12.7)	56.0 (12.0)	55.3 (13.0)
Rescue salbutamol, puffs/day, mean (SD)	2.2 (2.5)	2.7 (2.9)	2.0 (2.3)
GOLD spirometric grade^b^, n (%)			
2	1569 (65)	416 (67)	1153 (64)
3	851 (35)	207 (33)	644 (36)
% reversibility to salbutamol, mean (SD)	10.5 (13.1)	26.2 (13.3)	5.0 (7.4)
CAT score, mean (SD)	19.2 (6.0)	20.0 (6.5)	18.9 (6.0)
BDI score, mean (SD)	7.0 (1.9)	7.0 (2.0)	7.0 (1.8)
E-RS total score	10.6 (5.7)	11.7 (5.9)	10.2 (5.6)
SGRQ score, mean (SD)	44.7 (16.2)	46.3 (17.3)	44.1 (15.7)

Reversibility was defined as an increase in post-salbutamol FEV_1_ of ≥ 12% and ≥ 200 mL; ^a^number of exacerbations requiring oral or systemic corticosteroids and/or antibiotics (moderate) in 12 months prior to screening (patients with > 1 moderate exacerbation or with a severe exacerbation [requiring hospitalisation] were excluded); ^b^an additional 4 (< 1%) patients with GOLD grade 1 were randomised (reversible n = 2; non-reversible n = 2)

BDI, Baseline Dyspnoea Index; CAT, COPD Assessment Test; COPD, chronic obstructive pulmonary disease; E-RS, Evaluating Respiratory Symptoms-COPD; FEV_1_, forced expiratory volume in 1 s; GOLD, Global Initiative for Chronic Obstructive Lung Disease; ITT, intent-to-treat; SD, standard deviation; SGRQ, St George’s Respiratory Questionnaire

The majority of patients (2202/2425 [91%]) had a reversibility between -100 and 400 mL (Fig. 1), therefore the FP plots show data for this range.

### FP analyses

#### Lung function

**Fig. 2:**
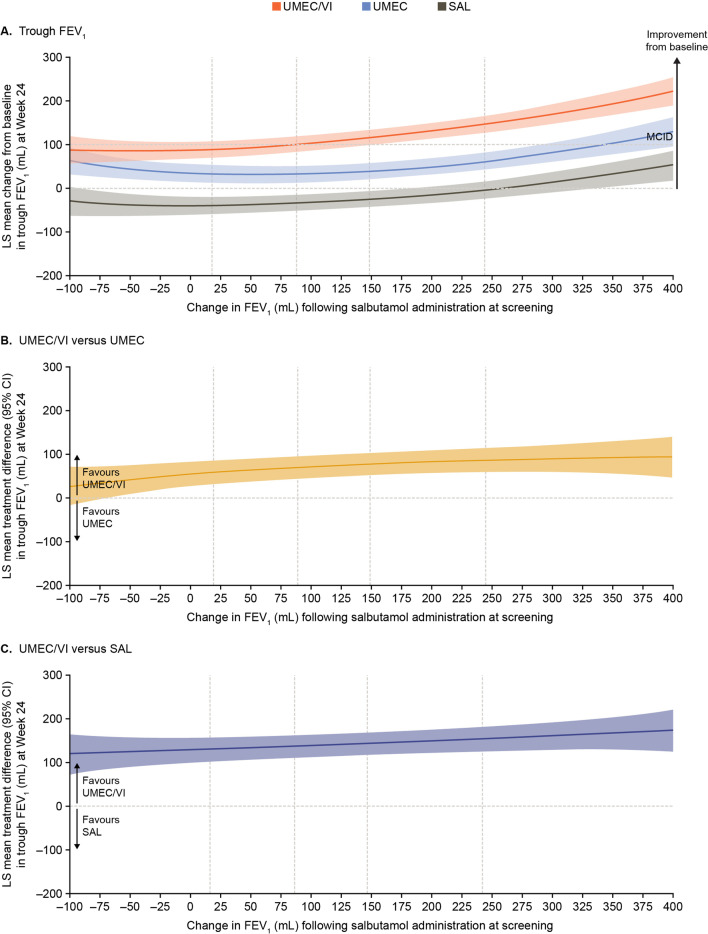
Improvement in trough FEV_1_ at Week 24. Vertical dotted lines indicate quintiles of reversibility at baseline. The MCID is ≥ 100 mL increase of trough FEV_1_ from baseline [12]. CI, confidence interval; FEV_1_, forced expiratory volume in 1 s; FP, fractional polynomial; LS, least squares; MCID, minimal clinically important difference; SAL, salmeterol; UMEC, umeclidinium; VI, vilanterol

At Week 24, as reversibility levels at screening increased, the adjusted mean change from baseline in trough FEV_1_ also increased in a similar way in all treatment arms (Fig. 2A). Using the chosen model, the lower bound of the 95% CI excluded 0 for UMEC/VI and UMEC, but not for SAL. The lower bound of the 95% CI for the mean change from baseline with UMEC/VI exceeded the 100 mL minimal clinically important difference (MCID) in trough FEV_1_ for reversibility > 175 mL, but did not exceed the MCID with either monotherapy. Consistent improvements in trough FEV_1_ were observed with UMEC/VI versus UMEC and SAL across the range of reversibility at screening, with a trend towards greater treatment differences at the higher values of reversibility (Fig. 2B and C).

#### SAC-TDI

**Fig. 3:**
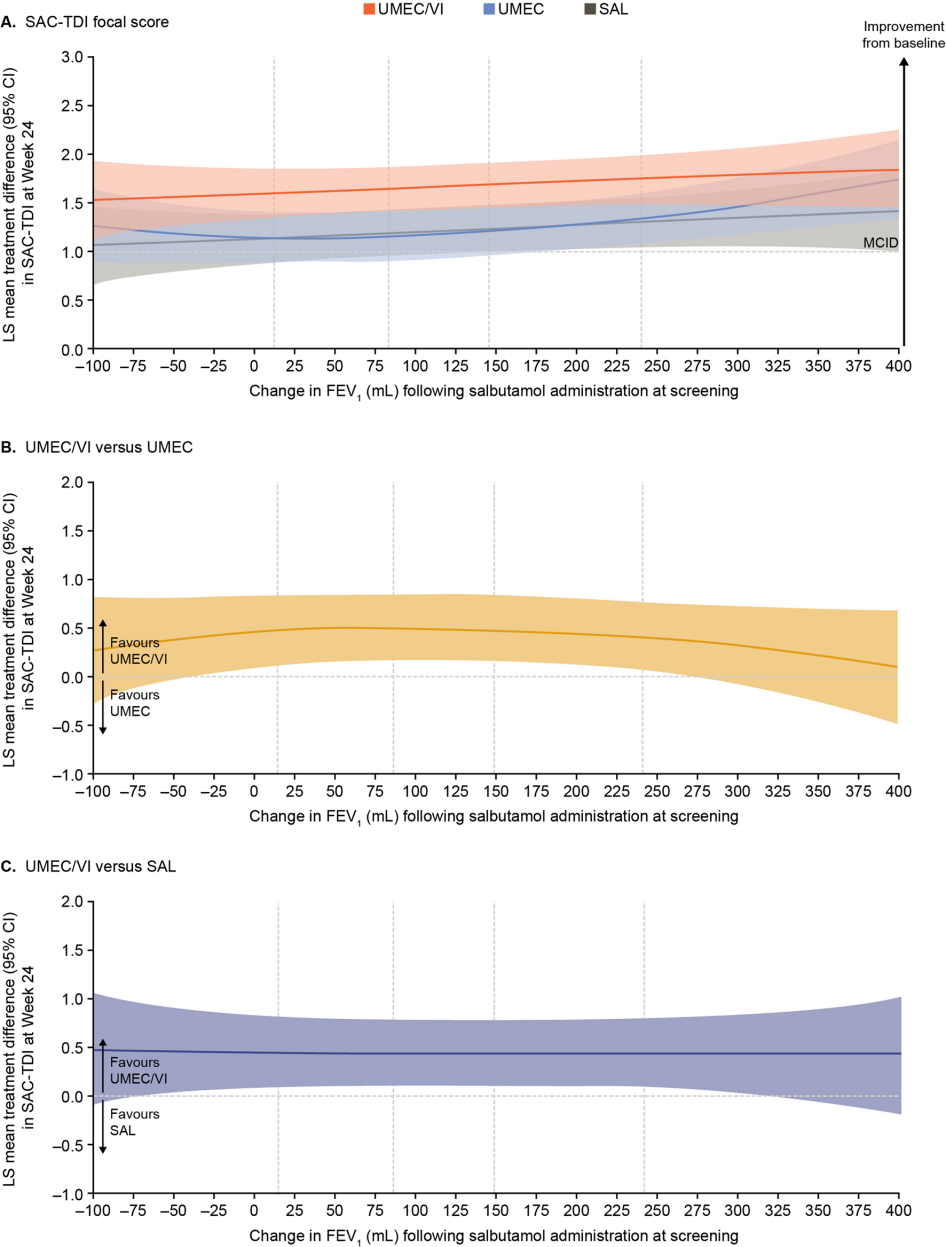
Improvement in SAC-TDI focal score at Week 24. Vertical dotted lines indicate quintiles of reversibility at baseline. The MCID of SAC-TDI score is ≥ 1-point improvement from baseline [13]. BDI, Baseline Dyspnoea Index; CI, confidence interval; FEV_1_, forced expiratory volume in 1 s; FP, fractional polynomial; LS, least squares; MCID, minimal clinically important difference; SAC-TDI, self-administered computerised-Transition Dyspnoea Index; SAL, salmeterol; UMEC, umeclidinium; VI, vilanterol

At Week 24, improvements in SAC-TDI score were similar for all treatments and the lower bound of the 95% CIs for these improvements excluded 0 across the full range of reversibility. The lower bound of the 95% CI for the mean improvement with UMEC/VI also exceeded the 1-point MCID across the full range of reversibility. There was a small trend towards greater improvements at higher levels of reversibility (Fig. 3A). The between-treatment differences in SAC-TDI favoured UMEC/VI versus UMEC and SAL within the mid-range of reversibility (Fig. 3B and C).

#### E-RS

**Fig. 4:**
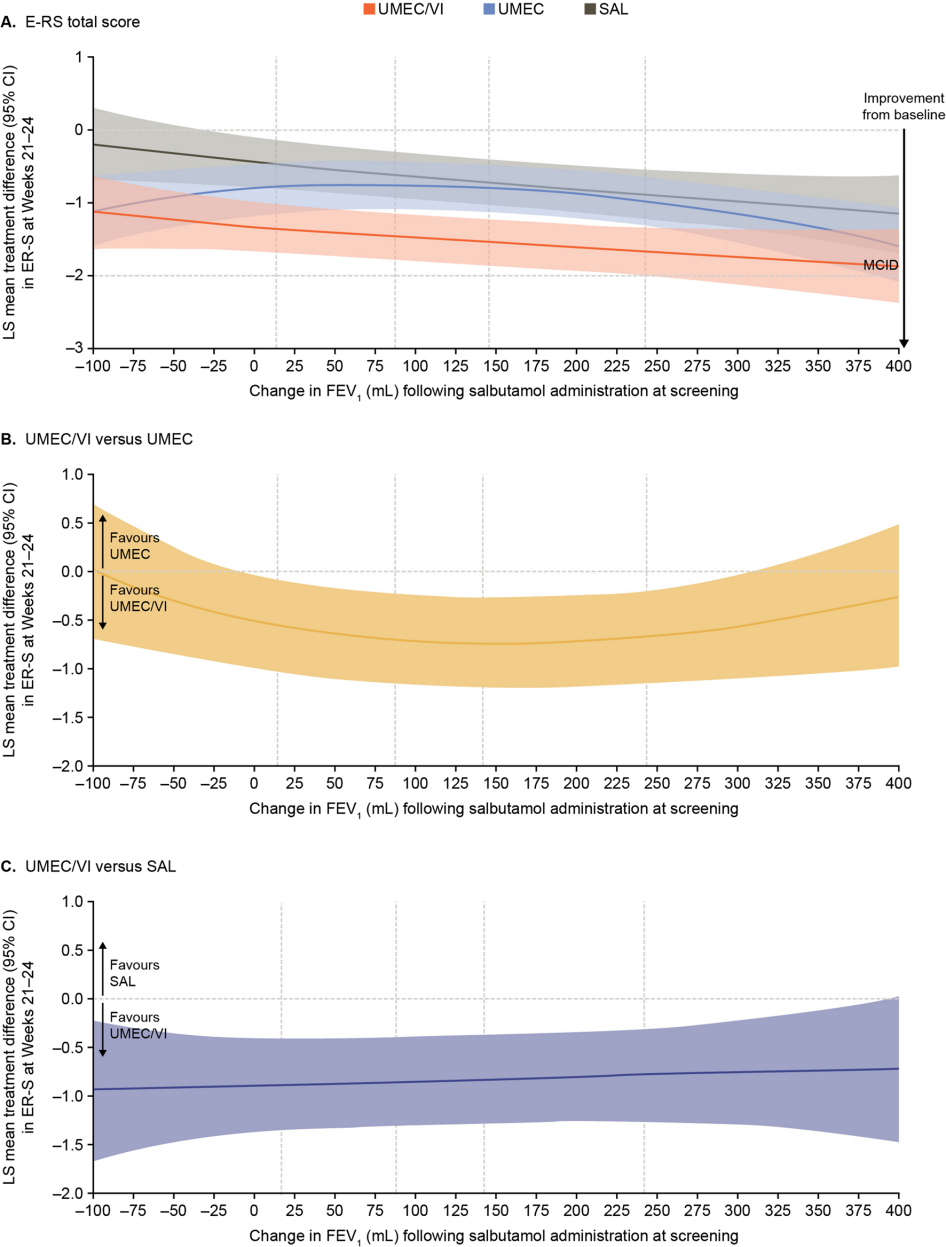
Improvement in E-RS total score at Weeks 21–24. Vertical dotted lines indicate quintiles of reversibility at baseline. The MCID of E-RS total score is a ≥ 2-point reduction from baseline [14]. CI, confidence interval; E-RS, Evaluating Respiratory Symptoms-COPD; FEV_1_, forced expiratory volume in 1 s; FP, fractional polynomial; LS, least squares; MCID, minimal clinically important difference; SAL, salmeterol; UMEC, umeclidinium; VI, vilanterol

At Weeks 21–24, improvements from baseline in E-RS total score were observed across the range of reversibility at screening for all treatments with the exception of SAL where the lower bound of the 95% CI included 0 at the lower values of reversibility. There was a trend towards greater improvements in E-RS with higher reversibility; however, the mean change from baseline did not reach the MCID (Fig. 4A). The between-treatment differences in E-RS total score favoured UMEC/VI in the middle of the reversibility range versus UMEC and across the full range of reversibility versus SAL (Fig. 4B and C).

### Rescue medication use

**Fig. 5:**
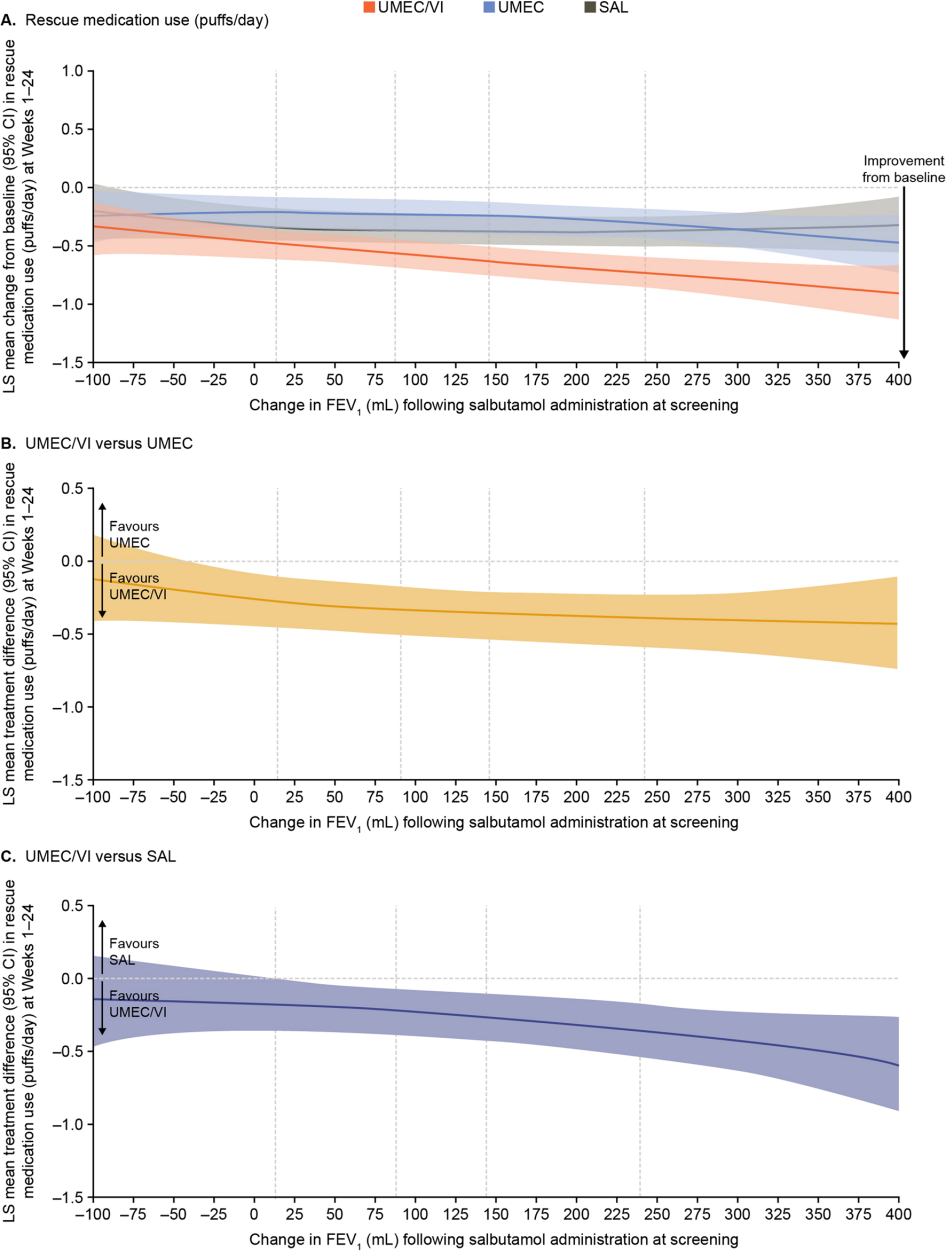
Improvement in rescue medication use (puffs/day) across Weeks 1−24. Vertical dotted lines indicate quintiles of reversibility at baseline. CI, confidence interval; FEV_1_, forced expiratory volume in 1 s; FP, fractional polynomial; LS, least squares; SAL, salmeterol; UMEC, umeclidinium; VI, vilanterol

The 95% CI for change from baseline in rescue medication use over Weeks 1–24 excluded 0 across all values of reversibility indicating improvement with all treatments irrespective of the degree of reversibility at screening (Fig. 5A). The between-treatment differences in rescue medication use favoured UMEC/VI versus UMEC and SAL across the majority of the range of reversibility. For both treatment comparisons there was a trend towards greater treatment differences for higher values of reversibility, which was more pronounced for UMEC/VI versus SAL than UMEC/VI versus UMEC (Fig. 5B and C).

### Interaction tests

From the MMRM, baseline reversibility was found to be a highly significant (*P* < 0.0001) covariate on trough FEV_1_. There is evidence to suggest (*P* = 0.0298) treatment has a different effect on trough FEV_1_ at different levels of baseline reversibility.

Baseline reversibility was found to be a significant covariate on SAC-TDI (*P* = 0.0452), E-RS total score (*P* = 0.0019) and rescue medication use (*P* = 0.0276). However, the models showed no evidence to suggest that treatment has an effect on SAC-TDI (*P* = 0.9032), E-RS total score (*P* = 0.4785) or rescue medication use (*P* = 0.1450) at different levels of reversibility.

## Discussion

This analysis of the EMAX trial found that greater reversibility at screening was generally associated with better treatment responses for all treatments. Patients receiving UMEC/VI had greater improvements in lung function, E-RS total score, and reduced medication use compared with patients receiving UMEC or SAL, irrespective of their degree of reversibility at screening.

The main findings of this analysis are concordant with other studies, such as a pooled analysis of the FLIGHT studies that demonstrated greater improvements in lung function and greater reductions in rescue medication use with the twice-daily LAMA/LABA indacaterol/glycopyrrolate compared with placebo in reversible versus non-reversible patients [7]. In the EMAX analyses, the association between the degree of reversibility and SAC-TDI was less clear; patients receiving UMEC/VI had greater improvements in SAC-TDI score versus patients receiving UMEC or SAL across a narrower reversibility range compared with other endpoints. Furthermore, the repeated measures analyses found no evidence to suggest an interaction between baseline reversibility and treatment for SAC-TDI. However, the trial was not powered to examine an interaction between baseline reversibility and the endpoints described. In the FLIGHT analyses, TDI scores in patients who were reversible and non-reversible were similar [7]. In contrast, analysis of the GEM studies found TDI scores were significantly greater with glycopyrrolate administered twice daily versus placebo in reversible patients but not in non-reversible patients; however, this could potentially be due to the higher TDI response in the non-reversible patients receiving placebo [6] and it should be noted that the subgroup sizes were small, with approximately 200 patients in each.

The efficacy of tiotropium compared with placebo has been demonstrated in patients categorised as responsive (improvement in FEV_1_ of ≥ 12% and ≥ 200 mL within 180 min of the first dose of tiotropium) and poorly-responsive to tiotropium [8]. At 1 year of treatment, both groups demonstrated significant improvements in lung function, SGRQ total score, TDI, and rescue medication use with tiotropium versus placebo. However, significantly greater improvements between responsive and poorly responsive patients were only observed for the TDI endpoint [8]. A longer-term analysis of patients in the 4-year UPLIFT study also demonstrated that irrespective of baseline reversibility, patients who received tiotropium had greater improvements in lung function compared with patients who received placebo. Similar to other studies, patients who were reversible at baseline had greater improvements in lung function compared with patients who were non-reversible [9].

This analysis of the EMAX trial provides important new information on the efficacy of LAMA/LABAs irrespective of reversibility because of the unique characteristics of the study population, which included ICS-free patients with symptomatic COPD. Pooled analyses of the GEM studies found that in patients treated with glycopyrrolate, improvements in trough FEV_1_ were significantly greater among reversible patients compared with non-reversible patients in a subgroup of patients who were ICS-free but not in patients who were receiving ICS [6]. This illustrates the importance of studying ICS-free COPD populations.

In EMAX, 26% of patients were reversible to salbutamol (a SABA) at screening, whereas in other studies, which focused on COPD populations who were permitted to use ICS, 49% of patients met reversibility criteria after SAMA and 52–55% were reversible to SAMA + SABA [6, 7, 9]. The mean baseline reversibility in EMAX was 10.5%, which was lower than baseline reversibility in the pooled analysis of the FLIGHT studies (22.8%) and the UPLIFT trial (23.4%) [7]. In addition to differences in study populations, it is important to note that differences in reversibility test protocols contribute to variability in which patients will be considered as meeting reversibility criteria, as there is currently no standardised procedure for assessing bronchodilator reversibility [2]. For instance, a study showed that assessing reversibility with a combination of ipratropium and salbutamol (SAMA/SABA) produced more consistent results versus either drug alone [11]. Key differences between the EMAX trial and the reversibility tests in previous studies were the drug used (a SABA vs a SAMA or a SAMA/SABA combination) and the absence of a wash-out of LABA or LAMA in EMAX. The use of a SAMA/SABA and/or a wash-out period will have contributed to the higher degree of reversibility in other studies. In clinical practice reversibility is commonly measured using a SABA and is conducted without a wash-out period, therefore, bronchodilator reversibility as assessed in the EMAX trial likely more closely emulates clinical practice.

Another interesting finding from this study is that despite the fall in trough FEV_1_ in the SAL-treated patients with lower bronchodilator reversibility, these patients have an improvement in symptoms, with a reduction in rescue medication use and an improvement in breathlessness. This suggests that trough FEV_1_ may not be the best spirometric correlate of breathlessness during daily activities and the need for rescue medication, both of which are reported later in the day after the morning dose of bronchodilator has been taken, rather than first thing in the morning.

A strength of this analysis is that it allows modelling of the relationship between reversibility as a continuous variable and other outcomes and avoids the need for any arbitrary pre-defined cut-off values. Most patients had reversibility between −100 and 400 mL; however, at the extreme ends of the reversibility range shown in the FP plots, there are fewer patients, therefore the CIs are wider and the ability to discriminate between treatments is diminished. Another strength is that the EMAX trial comprised 24 weeks follow-up of patients with COPD with a low risk of exacerbations not receiving ICS, and included maintenance treated and naïve patients, therefore, it provides new data to support the optimal treatment of these patient groups.

A limitation of this study is that only one measurement of reversibility was taken at screening, and variation in reversibility measurements have been previously reported [2, 11]. In addition, bronchodilator reversibility can be assessed using either a SABA, a SAMA or a combination of both, and in this study reversibility measurements were taken with only one agent (a SABA). Furthermore, we are not able to assess whether reversibility may correlate with exacerbation risk, as the EMAX trial investigated patients with a low exacerbation risk and few exacerbations occurred during the trial. Finally, the FP analyses should be interpreted with caution as a best fitting model was used and the CIs do not take in to account uncertainty in the model selected.

## Conclusions

FP modelling suggests that in symptomatic patients with COPD not receiving ICS, patients with higher levels of reversibility have greater improvements in lung function and symptoms in response to long-acting bronchodilators. The improvements in all outcomes were generally greater with UMEC/VI versus monotherapy independent from the degree of reversibility, suggesting that the dual bronchodilator UMEC/VI may be appropriate treatment for patients with symptomatic COPD irrespective of short-term response to bronchodilation. Sufficiently powered prospective studies are required to support these findings.

## Supplementary Information

**Additional file 1.** Plain language summary.

**Additional file 2.** Independent Ethics Committee/Institutional Review Board that approved the EMAX trial.

## Data Availability

Anonymised individual participant data and study documents can be requested for further research from www.clinicalstudydatarequest.com.

## Abbreviations

ATS: American Thoracic Society
CAT: COPD Assessment Test
CI: Confidence interval
COPD: Chronic obstructive pulmonary disease
ERS: European Respiratory Society
E-RS: Evaluating Respiratory Symptoms-COPD
FEV_1_: Forced expiratory volume in 1 s
FP: Fractional polynomial
GOLD: Global Initiative for Chronic Obstructive Lung Disease
ICS: Inhaled corticosteroid
IQR: Interquartile range
ITT: Intent-to-treat
LABA: Long-acting β_2_-agonist
LAMA: Long-acting muscarinic antagonist
LS: Least squares
MCID: Minimal clinically important difference
MMRM: Mixed model repeated measures
SAC-TDI: Self-administered computerised-Transition Dyspnoea Index
SAL: Salmeterol
SD: Standard deviation
SABA: Short-acting β_2_-agonist
SGRQ: St George’s Respiratory Questionnaire
SAMA: Short-acting muscarinic antagonist
UMEC: Umeclidinium
VI: Vilanterol
