# Supplementary material for: Efficacy of umeclidinium/vilanterol according to the degree of reversibility of airflow limitation at screening: a post hoc analysis of the EMAX trial
Source: Respir Res. 2021 Oct 28;22:279. doi: 10.1186/s12931-021-01859-w (PMC8555352; doi:10.1186/s12931-021-01859-w)
Supplement: Supplementary file 1 — Additional file 1. Plain language summary. [file 12931_2021_1859_MOESM1_ESM.docx]

## Additional File 1

**Plain language summary**

Patients with chronic obstructive pulmonary disease (COPD) have narrowed airways that cause airflow to be obstructed making it difficult for them to breathe out. COPD can be treated with bronchodilators, medicines which open the airways and make it easier for patients to breathe. Reversibility of airflow limitation is a measure of how much improvement there is in airflow shortly after treatment with a short-acting bronchodilator. The EMAX clinical trial examined potential benefits of a combination of two bronchodilators with different mechanisms of action (umeclidinium/vilanterol) compared with a single bronchodilator (either umeclidinium or salmeterol) in patients with symptomatic COPD over a period of 6 months. In this study, we measured bronchodilator reversibility in patients before they were given any study treatment, to assess if the level of reversibility can be used to determine which patients will benefit from dual therapy rather than monotherapy. Overall, we found that after 6 months of treatment, patients had improvements in their lung function and symptoms regardless of their level of reversibility before they started the study treatment. Patients treated with umeclidinium/vilanterol had greater improvement in lung function and were less dependent on their rescue inhaler compared with patients treated with umeclidinium or salmeterol. We conclude that the dual bronchodilator umeclidinium/vilanterol may be an appropriate treatment for patients with symptomatic COPD, regardless of their level of reversibility with a short-acting drug.
