# Supplementary material for: Efficacy of umeclidinium/vilanterol according to the degree of reversibility of airflow limitation at screening: a post hoc analysis of the EMAX trial
Source: Respir Res. 2021 Oct 28;22:279. doi: 10.1186/s12931-021-01859-w (PMC8555352; doi:10.1186/s12931-021-01859-w)
Supplement: Supplementary file 2 — Additional file 2. Independent Ethics Committee/Institutional Review Board that approved the EMAX trial. [file 12931_2021_1859_MOESM2_ESM.docx]

## Additional File 2

**Independent Ethics Committee/Institutional Review Board that approved the EMAX trial**

| Comité de Ética en Investigación. INAER, Arenales 3146 1° B, Ciudad Autónoma de Buenos Aires, C1425BEN, Argentina |
| --- |
| CEICI, Italia 424, Rosario, Santa Fe, 2000, Argentina |
| CEMER, Esmeralda 1550, Florida, Buenos Aires, 1602, Argentina |
| CECIC Comité de Ética de CER Investigaciones Clínicas, Vicente Lopez 1441, Quilmes, Buenos Aires, 1878, Argentina |
| Comite Independiente de etica Fundacion Rusculleda, Avenida Colon 2057, Cordoba, Córdoba, X5003DCE, Argentina |
| Framingham Centro Médico, Calle 9 Numero 431, La Plata, Buenos Aires, B1902COS, Argentina |
| Comité Independiente de ética para ensayos en Farmacología Clinica del centro Medico Dra. De Salvo, Avenida Cabildo 1536 5° B, Ciudad Autonoma de Buenos Aires, Buenos Aires, C1426ABP, Argentina |
| Instituto Argentino de Investigacion Neurologica, Uruguay 824 1st floor, Ciudad Autonoma de Buenis Aires, C1015ABR, Argentina |
| FUMELIT, Av. Gdor. Freyre 3074, Santa Fe, 3000, Argentina |
| CIDEA, 3er Cuerpo - 2do Subsuelo, Paraguay 2035, Ciudad Autónoma de Buenos Aires, C1121ABE, Argentina |
| Comite de Etica de San Isidro-CESI, Avenue Libertador 169581, San Isidro, Buenos Aires, CP 1643, Argentina |
| Comite de etica para la investigación Clinica Fundación Dr. J.R. Villavicencio, Alvear 854, Rosario, Santa Fe, 2000QGB, Argentina |
| Comite de Etica Iniciativa y Refelexion Bioetica Rosario, Rioja 2926, Rosario, S2002OJN, Argentina |
| CESIM, Urquiza 646, Santa Rosa, 6300, Argentina |
| Instituto de Investigacion Clinica de Mar del Plata, Av. Colon 3364 PB, Mar del Plata, Buenos Aires, B7600FZN, Argentina |
| Centro de Osteopatias Medicas, Azcuenaga 1860, Buenos Aires, C1128AAF, Argentina |
| Comite de Etica en Investigacion Clinica - CEIC, Larrea 1381, Ciudad Autonoma de Buenos Aires, Buenos Aires, C1117ABK, Argentina |
| Comite de Etica en Investigacion, Instituto Ave Pulmo, Carlos M. Alvear 3345, Mar del Plata,  Buenos Aires, B7602DCK, Argentina |
| Bellberry Limited, 129 Glen Osmond Rd, Eastwood, South Australia, 5063, Australia |
| INSTITUTIONAL REVIEW BOARD Services, Suite 300, 372 Hollandview Trail, Aurora, Ontario, L4G 0A5, Canada |
| Comité d'éthique de la recherche de l'Institut universitaire de cardiologie et de pneumologie de Qué, 2725 Chemin Ste-Foy, Quebec, G1V 4G5, Canada |
| CHU Pontachaillou, Comité de Protection des Personnes, 9 Avenue de la Bataille Flandres-Dunkerque Mai 1940, Rennes, 35000, France |
| Ethik-Kommission der Landesaerztekammer Hessen, Im Vogelsgesang 3, Frankfurt, Hessen, 60488, Germany |
| Comitato Etico IRCCS Istituto Tumori "G.Paolo II", V.le Orazio Flacco 65, Bari, Puglia, 70124, Italy |
| Comitato Etico Campania Nord c/o A.O. San Giuseppe Moscati di Avellino, Segreteria Scientifico- Amministrativa, Città Ospedaliera -Pal. Uffici, Contrada Amoretta, Avellino, Campania, Italy |
| Com. Etico Reg. Toscano "Area Vasta Nord Ovest", Segreteria Scientifico-Amministrativa -Azienda Ospedaliero-Universitaria Pisana, Via Roma, 67, Pisa, Toscana, 56126, Italy |
| Comitato Etico Palermo 1, c/o Azienda Ospedaliera Universitaria Policlinico "Paolo Giaccone", Segreteria Scientifico-Amministrativa, Via del Vespro 129, Palermo, Sicilia, 90127, Italy |
| Comitato Etico Univ. Studi Campania L.Vanvitelli – AOU L.Vanvitelli - AORN Osp. dei Colli, Sede AORN Ospedali dei Colli, Via Leonardo Bianchi snc, Napoli, Campania, 80131, Italy |
| C.E.ROM. Comitato Etico della Romagna, c/o Ist. Scientifico Romagnolo per lo Studio e la Cura dei Tumori, IRST - IRCCS Srl, Via Piero Maroncelli 40, Meldola (FC), Emilia-Romagna, 47014, Italy |
| Comitato Etico Unico Regionale del Friuli Venezia Giulia, Segreteria Tecnico-Scientifica c/o Dir. Scientifica, IRCCS CRO di Aviano, Via Gallini, 2, Aviano (PN), Friuli-Venezia-Giulia, 33081, Italy |
| Comitato Etico dell'Area Vasta Emilia Nord, Via Vertoiba, 10A, Reggio Emilia, Emilia-Romagna,  42124, Italy |
| Comitato Etico Aziende Sanitarie dell'Umbria, Segreteria Scientifico-Amministrativa, Via della Rivoluzione, 16, Ellera di Corciano (PG), Umbria, 6070, Italy |
| Comitato Etico Area 4 – ASL Brindisi, Segreteria Scientificoamministrativa, Via Napoli, 8, Brindisi, Puglia, 72100, Italy |
| Comitato Etico Univ. Studi Campania L.Vanvitelli – AOU L.Vanvitelli - AORN Osp. dei Colli, Sede AORN Ospedali dei Colli, Via Leonardo Bianchi snc, Napoli, Campania, 80131, Italy |
| Comitato Etico degli Istituti Clinici Scientifici Maugeri SpA – SB, Via Salvatore Maugeri 4, Pavia, Lombardia, 27100, Italy |
| Comitato Etico Campania Sud c/o ASL Napoli 3 Sud, Segreteria Scientifico-Amministrativa, Piazza San Giovanni, 7, Brusciano (NA), Campania, 80031, Italy |
| Hospital Real San Jose, Av Lázaro Cárdenas 4149 Colonia Jardines de San Ignacio, Zapopan, Jalisco, 45040, Mexico |
| Instituto Jalisciense de Investigación Clínica, S.A. de C.V., Penitenciaria 20, Guadalajara, Jalisco, 44100, Mexico |
| St. Antonius Ziekenhuis, Koekoekslaan 1, NIEUWEGEIN, 3435 CM, Netherlands |
| Pharma Ethics, 123 Amcor Road, Lyttelton Manor, 157, South Africa |
| University of Cape Town, Human Research Ethics Committee, Room E52.24, Old Main Building, Groote Schuur Hospital, Main Road, Observatory, 7925, South Africa |
| Fundació Unió Catalana d’Hospitals, 1ºA, C/Bruc 72-74, Barcelona, 8009, Spain |
| Regionala Etikprövningsnämnden I Göteborg, Guldhedsgatan 5A, GÖTEBORG, SE-413 20, Sweden |
| Advarra Institutional Review Board, Suite 110, 6940 Columbia Gateway Drive, Columbia, Maryland, 21046, United States |
| Saint Luke’s Hospital Institutional Review Board, 232 South Woods Mill Road, Chesterfield, Missouri, 63017, United States |
| South Carolina Pharmaceutical Research, 141 Harold Fleming Court, Spartanburg, South Carolina, 29303, United States |
